# West Country Physicians Club, Spring Meeting 1982

**Published:** 1982

**Authors:** 


### West Country Physicians' Club – Spring Meeting 1982

The Club held its 70th meeting at the Postgraduate Medical Centre, Torbay Hospital, on Friday and Saturday, 2nd and 3rd April, 1982. This was the last occasion on which Bill Barritt would have attended, but he was elected an honorary member and we shall look forward to seeing him at future meetings. His health and that of Mrs. Barritt was toasted at the Club dinner at the Imperial Hotel. As usual Dr. M. G. Thorne and his colleagues managed to organise a most successful meeting, and somehow he and the drug companies made the eventual cost extremely modest!

Three new members were elected: Dr. Roger Corrall (General Medicine, Endocrinology & Diabetes, Bristol), Dr. Andrew Marshall (General Medicine & Cardiology, Plymouth), and Dr. Robin Teague (General Medicine & Gastroenterology, Torbay). Surgeon Commander John Williams RN was also elected for the duration of his stay at the Naval Hospital, Plymouth.

At the business meeting held on Saturday morning, it was agreed that those who gave papers in the session of Scientific Commentaries should be invited to submit a summary for inclusion in the *Bristol Med Chi Journal.* Of the six papers given in the first session on Saturday morning, four are presented in that form below. The six papers given were:

Angiodysplasia of the colon
Dr. R. H. Teague
Immune complexes in early arthritis
Dr. R. K. Jacoby
Diamorphine by continuous intravenous drip in myocardial infarction
Dr. M. G. Thorne
A new tubeless test of pancreatic exocrine function
Dr. R. E. Barry
Natural history of Cushing's Syndrome
Prof. D. Mattingly
Low renin cirrhosis
Dr. S. P. Wilkinson

Members of the Club are reminded that the next meeting – the 71st – will be held in Cheltenham on 5th and 6th November. May we all congratulate Torbay members for putting on such an excellent meeting for us all.

A.E.R.

### LOW/NORMAL RENIN CIRRHOSIS

**S. P. Wilkinson**
**(Gloucestershire Royal Hospital**

The renin-angiotensin-aldosterone (RAA) system is generally thought to be stimulated in cirrhosis especially when ascites is present, but recent studies have not usually confirmed this concept.[1] In a group of patients avidly retaining sodium and accumulating ascites only one-third had increased plasma levels for the components of the system, normal values being found in the others. For patients without clinical evidence of fluid retention the levels were reduced to one-half of those found for healthy control subjects. This suppression of the system may have been secondary to expansion of the extracellular and blood volumes already well documented at this stage of cirrhosis.

The normal aldosterone levels for patients retaining sodium questions its pathogenic role. However, in the complete series sodium excretion was closely related to the plasma aldosterone concentration ($r = —0.794$, $p<0.001$). Other evidence suggesting a dominant role for aldosterone includes both the effect of ß-adrenergic blockade in which sodium excretion changed exactly as predicted by the changes in aldosterone and the effect of spironolactone in reversing the sodium retention provided enough is given.

These findings could all be explained if there is an increased renal tubular sensitivity to aldosterone in cirrhosis. This might be an early abnormality present before ascites is detectable and result in the well documented clinically undetectable volume expansion, with consequent suppression of the RAA system. At a more advanced stage of the disease a higher portal and reduced oncotic pressures would result in the retained fluid "overflowing" to the peritoneal cavity as ascites, the "effective" extracellular volume therefore not being expanded and sodium retention continuing with normal aldosterone levels.

[1]Wilkinson, S. P., Williams, R. Renin-angiotensin-aldosterone system in cirrhosis. Gut, 1980, 21: 545–554.

### THE RELIEF OF PAIN IN ACUTE MYOCARDIAL INFARCTION BY CONTINUOUS INTRAVENOUS INFUSION OF DIAMORPHINE

**G. Thommi and M. G. Thorne**
**(Torbay Hospital, Torbay)**

A trial to test the efficacy of Diamorphine given by continuous intravenous infusion in acute myocardial infarction, was planned as follows. Patients were randomly allocated to two groups:

(A) Diamorphine 0.075 mg – 0.15 mg/kg I.M. stat and four hourly or earlier as required.
(B) Diamorphine 2 mg I.V. stat and then as an infusion of 0.03 mg/kg per hour. The rate was doubled after 15 minutes if necessary, reducing later with pain relief. It was halved after two hours and stopped after four hours in absence of pain.

Patients were readmitted to original schedules if pain recurred more than four hours after initial relief.

Blind assessment was by independant nurses two hourly, using four grades: (1) Pain free; (2) Pain but able to rest; (3) Pain disturbing rest; (4) Severe pain. Respiratory rate, blood pressure, heart failure, cardiac enzymes, anti-emetic requirements were also recorded.

48 patients in group A and 42 in group B were comparable in age, sex, previous infarction history, infarct site and pain intensity. Group A contained eight diabetics, group B three.

### RESULTS

#### Pain Assessment

| | Pain Grade On entry | Pain Grade Average through-out trial | Pain Duration in hours | Grade X Duration | Patients re-entering trial |
|---|---|---|---|---|---|
| A | 2.8 | 1.5 | 6.75 | 10.1 | 7 |
| B | 2.9 | 1.4 | 5.25 | 7.35 | 5 |

#### Other Clinical Features

| | Deaths | Ventricular Arrhythmias | Patients requiring Anti-emetics | Average serum enzyme levels SGOT | Average serum enzyme levels LDH | Heart Failure |
|---|---|---|---|---|---|---|
| A | 3 | 5 | 24 (50%) | 234 | 1848 | 22 |
| B | 5 | 3 | 13 (35%) | 174 | 1486 | 15 |

Blood pressures and respiratory rates were not significantly different in the two groups.

#### Diamorphine Dosage

| | Average total mgs. | Dosage related to initial pain grade IV | III | II |
|---|---|---|---|---|
| A | 10.2 | 11.6 | 9.9 | 9.8 |
| B | 12.8 | 16.7 | 12.7 | 10.6 |

### CONCLUSIONS

Schedule B was:

(1) More effective in relieving the pain of myocardial infarction.
(2) More adaptable to individual patients' needs.
(3) Less nauseating.

### ANGIODYSPLASIA OF THE COLON

**R. H. Teague**
**(Torbay Hospital, Torquay)**

Vascular abnormalities of the large bowel were found in 34 of 902 cases of barium enema negative rectal bleeding at colonoscopy. The commonest types of abnormalities were flat mucosal ectasias which accounted for 76% of cases but polypoid angiomas, arteriovenous malformations, hereditary haemorrhagic telangectasias and colonic varicosities were also seen. The caecum and ascending colon were the usual sites of angiodysplasia but the condition was found at all other large bowel sites.

Almost all cases presented with brisk intermittent rectal bleeding and the mean haemoglobin at presentation was 7.1GMS. The average age at presentation was 65 years (range 8–88 years) and it was thought that the preponderance of elderly patients suggested an acquired lesion possibly secondary to an anoxic stimulus. In almost one-third of the patients there was a definite cardiac abnormality in the form of valvular heart disease, ischaemic heart disease or cor pulmonale. In younger patients the condition was almost certainly congenital in origin and these included the cases of hereditary haemorrhagic telangectasia.

Endoscopic treatment with "hot" biopsy or water-jet coagulation was successful in 27 of the 34 cases, four cases requiring further coagulation procedures at subsequent endoscopies. It is concluded that angiodysplasia is a significant cause of rectal bleeding and that endoscopic treatment is simple and successful in most cases.

### IMMUNE COMPLEXES IN EARLY ARTHRITIS

**Dr. Richard Jacoby and Dr. Valerie Jones**
**Post Graduate Medical Institute, University of Exeter**

Patients presenting within a few months of the onset of their arthritic symptoms were studied. The clinical progress of the patients was followed, 27 developed rheumatoid arthritis (R.A.) as defined by the American Rheumatism criteria. Of the patients who failed to convert into R.A. (henceforth called I.A. – inflammatory arthritis) within three years, a similar number of patients were selected to age and sex match the R.A. patients. Immune complexes were measured using platelet aggregation titres (P.A.T.) and C1q binding techniques. Rheumatoid factors were also measures.

**Results**
Sequential studies showed that both R.A. and I.A. patients developed P.A.T. complexes early on in the course of the arthritis. C1q binding complexes developed in all the R.A. patients and only one-third of the I.A. patients, in whom the titres were lower than the R.A. group. The appearance of immune complexes preceded the presence of rheumatoid factors in the R.A. patients.

**Comment**
This study suggests that a trigger factor gives rise to arthritis and in some individuals this develops into R.A., whereas I.A. patients arrest the disease. The genetic predisposition of the R.A. group to develop the disease is supported by the finding of an excess frequency of HLA DR4[1], in contrast to the I.A. group that has the expected distribution of antigens seen in the normal population.

[1]Panayi, G. S. et al. *B.M.J.* (1978) *2* 1326.

